# Expression and diagnostic values of ferroptosis-related genes in coronavirus-associated viral sepsis

**DOI:** 10.3389/fmed.2025.1496834

**Published:** 2025-04-30

**Authors:** Yidan Zhang, Shaoduo Wu, Xiaoyan Li

**Affiliations:** ^1^Department of Respiratory and Critical Care Medicine, Third Hospital of Shanxi Medical University, Shanxi Bethune Hospital, Shanxi Academy of Medical Sciences, Tongji Shanxi Hospital, Taiyuan, Shanxi, China; ^2^Department of Pulmonary Critical Care Medicine, Zhoupu Hospital in Pudong New Area, Shanghai University of Medicine and Health Sciences Affiliated Zhoupu Hospital, Shanghai, China

**Keywords:** ferroptosis, coronavirus, viral sepsis, IL1-β, HMOX1

## Abstract

**Aim:**

The aim of this study is to investigate the differential expression and diagnostic value of ferroptosis-related genes in coronavirus-associated viral sepsis.

**Methods:**

This study was conducted in two sequential phases: (1) identification of differentially expressed genes through comprehensive analysis of the experimental dataset (GSE164805); and (2) clinical validation of the candidate molecular markers using both test set samples and clinical samples, followed by rigorous evaluation of their diagnostic performance. Firstly, the microchips associated with coronavirus-associated viral sepsis were retrieved from the GEO database, a public data platform of NCBI (National Center for Biotechnology Information), and differentially expressed genes (DEGs) were obtained through differential analysis. The identified DEGs were then intersected with the ferroptosis gene dataset to obtain ferroptosis-related DEGs. Subsequently, molecular signaling pathways of ferroptosis-related genes in coronavirus-associated viral sepsis were analyzed using gene ontology (GO) and Kyoto Encyclopedia of Genes and Genomes (KEGG) enrichment analysis. CIBERSORT was employed to analyze immune cell infiltration in both the coronavirus-associated viral sepsis group and control group. Furthermore, a protein–protein interaction (PPI) network was constructed to identify hub genes involved in ferroptosis. Finally, the expression of ferroptosis hub genes in coronavirus-associated viral sepsis and its diagnostic value were analyzed in validation set GSE199816 and clinical case samples.

**Results:**

In test set GSE164805, a total of 15,059 differentially expressed genes (DEGs) were identified, comprising 7,458 up-regulated and 7,601 down-regulated genes. Subsequently, an intersection analysis with the ferroptosis gene dataset yielded 189 DEGs associated with ferroptosis. Functional enrichment analyses using GO and KEGG revealed significant enrichment in signaling pathways related to ferroptosis, oxidative stress, and HIF-1. Additionally, CIBERSORT immune-infiltration analysis revealed enhanced infiltration of innate immune cells but reduced infiltration of CD8^+^ T cells and natural killer (NK) cells in the coronavirus-associated viral sepsis group compared with healthy controls. Furthermore, analysis identified that the distribution of these immune cells correlated with the expression levels of IL1-β and HMOX1, suggesting that viral infection in the septic pathological state disrupts the balance between immune activation and suppression. Notably, PPI network analysis also identified IL1-β and HMOX1 as hub genes involved in ferroptosis. Finally, the results were verified in the validation set and clinical case samples, and the results showed that the expressions of IL1-β and HMOX1 in the coronavirus-associated viral sepsis group were decreased compared with the case control group and the healthy control group. In clinical samples, the expression levels were as follows: IL1-β (0.390 ± 0.068 vs. 1.101 ± 0.107), HMOX1 (0.629 ± 0.117 vs. 1.101 ± 0.107), and the differences were statistically significant (all *p* < 0.05). Further diagnostic performance analysis demonstrated that IL1-β and HMOX1 exhibited AUROCs of 0.892 and 0.765, respectively, indicating their robust diagnostic potential for coronavirus-associated viral sepsis.

**Conclusion:**

The present study has successfully identified two hub genes, IL1-β and HMOX1, associated with ferroptosis in coronavirus-associated viral sepsis, and their expression and diagnostic value for the disease. These findings provide effective diagnostic biomarkers and potential therapeutic targets for coronavirus-associated viral sepsis. Notably, this study specifically focused on coronavirus-induced viral sepsis, distinct from previously characterized bacterial sepsis and other viral etiologies, thus warranting future studies with expanded sample sizes for stratified analyses.

## 1 Introduction

Sepsis, a life-threatening organ dysfunction resulting from an aberrant host response to infection (1), afflicts millions of individuals worldwide annually, with mortality rates ranging from one sixth to one third (2), and has emerged as a significant public health concern. For an extended period, bacteria have been regarded as the primary pathogens while viral-induced infectious toxicities have been grossly underestimated on a global scale. However, it is noteworthy that patients with severe novel coronavirus pneumonia (COVID-19) frequently exhibit multi-organ functional impairments and those with viremia are at higher risk for organ injury (3). Present diagnostic and therapeutic strategies, encompassing antiviral agents and supportive care measures, possess notable limitations in effectively managing the disease particularly in severe cases (4). Consequently, there exists an urgent imperative for novel biomarkers and therapeutic targets aimed at enhancing patient outcomes.

The phenomenon of ferroptosis is a type of programmed cell death that occurs due to iron-dependent lipid peroxidation, which has garnered significant attention from researchers in the context of viral infection and sepsis (5). Previous studies have demonstrated differential expression of genes associated with ferroptosis in various diseases, including cancer and neurodegenerative disorders, suggesting their potential role in disease onset and progression (6). In the context of viral infections such as influenza and hepatitis, ferroptosis is believed to be linked to immune response regulation and tissue damage (7). However, the understanding of ferroptosis in the context of viral sepsis remains limited.

Based on this, the objective of this study was to investigate the molecular mechanism underlying iron-induced cell death in coronavirus-associated viral infection and identify potential biomarkers and therapeutic targets for improved patient outcomes. We proposed utilizing data from the GEO database^1^ for comprehensive data collection, analysis of hub genes, exploration of possible molecular mechanisms, and partial validation. Additionally, we aimed to screen and identify hub genes with potential diagnostic value for coronavirus-associated viral sepsis as well as potential therapeutic targets. These findings will serve as a foundation for accurate diagnosis and treatment of coronavirus-associated viral sepsis in clinical settings.

## 2 Materials and methods

### 2.1 Microarray dataset download and data preprocess

The microarray data set was screened from the GEO database (see text footnote 1) using the keywords “severe” and “viral infection “, resulting in the identification of GSE164805 (test set) and GSE199816 (validation set). The GSE164805 data set was generated using the GPL26963 platform, specifically the Agilent-085982 Arraystar Human lncRNA V5 microarray chip. Peripheral blood mononuclear cells (PBMCs) were collected from 5 patients with mild COVID-19, 5 patients with severe COVID-19, and 5 healthy controls. After standardization, annotation, and clearance of clinical information in dataset GSE164805, 5 samples from severe patients were selected as the coronavirus-associated viral sepsis group (test group) for bioinformatics analysis along with 5 samples from a healthy control group (control group). Dataset GSE199816 consisted of 9 patients with coronavirus-associated viral sepsis (test group) and 18 patients with bacterial sepsis (case control group). Ferroptosis-related genes including driver genes, suppressor genes, marker genes, and unclassified genes were obtained from FerrDb V2^2^ within its database.

### 2.2 Screening ferroptosis-related differentially expressed genes

First, we preprocessed the raw file obtained from the GEO database by removing probes associated with multiple molecules. In cases where a probe was associated with the same molecule, we selected the average value of the probes as the gene expression value. Differential expression genes (DEGs) were analyzed between the coronavirus-associated viral sepsis group and control group using the limma package in R software. The DEGs condition was set as follows: *p* < 0.05, |logFC| > 1. We obtained 564 ferroptosis genes from FerrDb V2 database. We performed an intersection analysis between DEGs and ferroptosis genes and visualized the results using Venn diagrams created with VennDiagram R package, heat maps created with Heatmap package, and volcano maps created with ggplot2 package.

### 2.3 Functional enrichment analysis

The gene ontology (GO) and the Kyoto Encyclopedia of Genes and Genomes (KEGG) functional enrichment analyses were conducted on the aforementioned differentially expressed genes (DEGs) associated with ferroptosis resulting from coronavirus-associated viral sepsis using the clusterProfiler R package (8). Gene ontological (GO) enrichment analysis is a widely used approach for investigating functional enrichments, including biological process (BP), cell component (CC), and molecular function (MF), and plays a crucial role in exploring biological functions (9). The Kyoto Encyclopedia of Genes and Genomes (KEGG) analysis is a network map that illustrates the relationships between individual genes or substances, aiming to explore potential signaling pathways (10).

### 2.4 Protein–protein interaction network analysis of ferroptosis-related DEGs

The evaluation and analysis of protein–protein interaction (PPI) networks are utilized to elucidate crucial interactions among key cellular genes (11). In this study, a PPI network analysis was constructed for the differentially expressed genes (DEGs) associated with ferroptosis caused by viral sepsis. This analysis was performed using the online tool STRING,^3^ which integrates known and predicted associations between proteins, including physical interactions and functional associations. Subsequently, the results from STRING were imported into Cytoscape, and the cytoHubba plug-in was used to download interaction data and enhance the PPI network to extract critical subnetworks. Finally, based on the maximum correlation criterion (MCC algorithm), we identified the hub gene as the one with the highest score within these subnetworks.

### 2.5 Evaluation and analysis of immune cell infiltration

This study utilized CIBERSORT (12) (Cell-type Identification By Estimating Relative Subsets Of RNA Transcripts), a deconvolution algorithm-based bioinformatics tool, to quantify the relative proportions of specific cell types from mixed tissue gene expression data. Through this methodology, we systematically evaluated immune cell infiltration in the samples, analyzing the distribution patterns of 22 immune cell subtypes (including CD8^+^ T cells, M1/M2 macrophages, neutrophils, and others). We further explored differences in immune cell marker expression between the coronavirus-associated viral sepsis group and healthy controls, thereby providing theoretical support for elucidating disease mechanisms and advancing precision therapeutics (13).

### 2.6 Collection of clinical specimens

The experimental group consisted of 20 patients hospitalized from June 2022 to December 2023 with coronavirus-associated viral sepsis, while the control group included 20 healthy adults who underwent physical examination at the same period in the physical examination center. Inclusion criteria: (1) Experimental group: I. Individuals who met the diagnostic criteria for viral sepsis; II. Aged ≥18 years old; III. Provided informed consent. (2) Control group: I. Individuals whose physical examination indicated good health; II. Aged ≥18 years old; III. Provided informed consent. Exclusion criteria: (1) Age <18 years; (2) Patients who died on admission or were transferred to hospitals with incomplete clinical data; (3) End-stage patients with chronic and malignant diseases; (4) Individuals who refused to participate in the research or sign informed consent; (5) People with a history of mental illness or cognitive dysfunction. This study was approved by the Clinical Research Ethics Committee of the hospital. Peripheral venous blood samples were collected from each subject, centrifuged at 4°C, and then stored immediately in a refrigerator at −80°C for future use. Table 1 shows the basic characteristics of the patients recruited from the control group and the test group.

**Table 1:** Comparison of baseline demographic characteristics and clinical parameters between patient group and healthy controls.

Indicators	Patients (*n* = 20)	Healthy people (*n* = 20)	*p*-value
Age, years	74.5(66.25–83.00)	74.5(70.25–76.75)	0.715
Men	12(60%)	11(55%)	0.749
Women	8(40%)	9(45%)	
Any comorbidity	10(50%)	0(0%)	
Hypertension	4(20%)	0(0%)	
Diabetes	3(15%)	0(0%)	
White blood cell count, 10^9^/L	7.60(5.30–10.10)	5.50(4.90–6.60)	0.083
Neutrophil count, 10^9^/L	5.50(1.41–9.30)	2.84(2.54–3.82)	0.095
Lymphocyte count, 10^9^/L	0.37(0.16–0.75)	1.78(1.32–2.09)	<0.001
Monocyte count, 10^9^/L	0.43(0.37–195)	0.46(0.37–0.53)	0.594

### 2.7 The mRNA expression of the hub genes of ferroptosis was determined by real-time PCR

The total RNA was extracted from the serum of the subjects. Reverse transcription was performed under the following conditions: 11 μL of RNA (1 μg) was mixed with 1 μL of random primer (0.2 μg/mL) and incubated at 65°C for 5 min. Subsequently, a mixture containing 4 μL of 5 × Buffer, 3 μL of dNTP (10 mmol/L), 1 μL of RNase inhibitor (20 U/uL), and 1 μL of reverse transcriptase (20 U/uL) was added to the reaction. The reaction proceeded at 25°C for 10 min, followed by incubation at 42°C for 1 h and finally at 72°C for 15 min. The real-time PCR reaction system consisted of 10 μL FastStart Universal SYBR Green Master (ROX), 0.5 μL upstream primer (15 μM), 0.5 μL downstream primer (15 μM), 2 μL cDNA, and 7 μL DNAse and RNase-free water, making a total volume of 20 μL. The primer sequence for human β-actin was as follows: upstream primer -GTCAGGTCATCACTATCGGCAAT-3′ and downstream primer -AGAGGTCTTTACGGATGTCAACGT-3′. Additionally, the ferroptosis hub gene primer sequence was included. PCR reaction conditions: The PCR reaction was initiated by pre-denaturation at 94°C for 10 min to activate the Tag enzyme. Subsequently, the amplification process consisted of denaturation at 94°C for 15 s, annealing at 60°C for 60 s, and a total of 40 cycles. The cycle threshold (CT) value was determined using fluorescence quantitative PCR. Firstly, the difference between the CT value of the target gene (iNOS) and that of the internal control gene Beta-actin in each sample was calculated as ΔCT = CT(iNOS)-CT(beta-actin). Then, ΔΔCT was obtained by subtracting the ΔCT value of samples from the experimental group with that from the normal control group. Finally, calculation using 2^(−ΔΔCT)^ indicated the fold change in expression of iNOS gene in relation to its expression in samples from normal control group. This analysis aimed to evaluate expression levels of hub genes associated with coronavirus-associated viral sepsis.

### 2.8 Statistical analysis

The data analysis was conducted using R software (version 4.2.3). Measurement data that followed a normal distribution were presented as $\bar{x}$ ±s. An independent sample T-test was employed to compare the two groups, while non-normally distributed data were expressed as M(P25, P75), and the Mann–Whitney U test was utilized for group comparisons. A significance level of *p* < 0.05 was considered statistically significant. Receiver Operating Characteristic (ROC) curve analysis was performed to evaluate the diagnostic efficiency of ferroptosis-related hub genes through sensitivity and specificity metrics, with the Area Under the Curve (AUC) serving as the core evaluation metric. Interpretation criteria were defined as follows: an AUC of 0.5 indicates no discriminative capacity (equivalent to random chance); 0.6–0.8 represents moderate discriminative power; 0.8–0.9 reflects high diagnostic accuracy; and values exceeding 0.9 demonstrate exceptional predictive performance (14).

## 3 Results

### 3.1 Identification DEGs between coronavirus-associated viral sepsis and control

**Figure 1:**
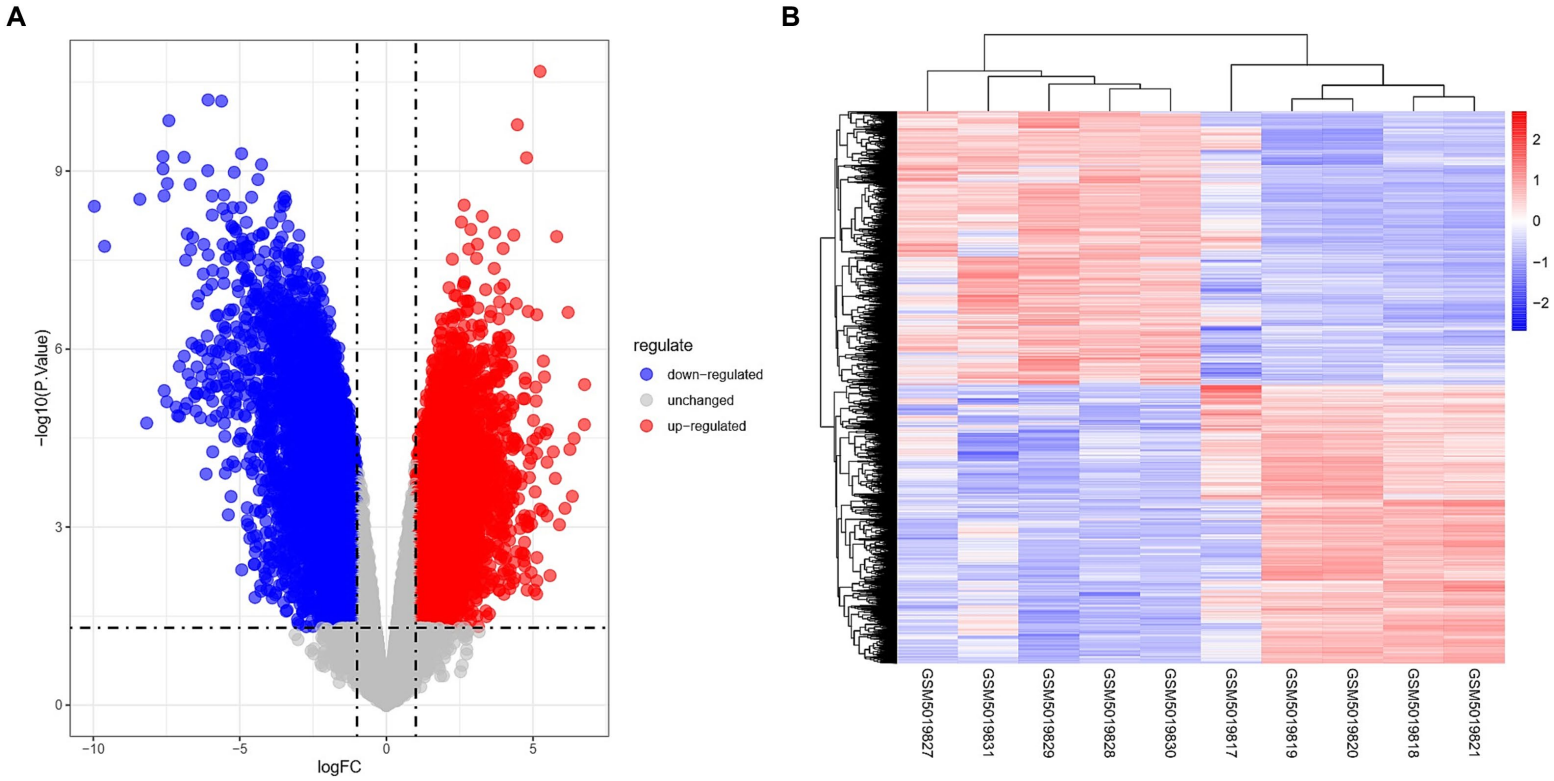
Overview of differentially expressed genes in coronavirus-associated viral sepsis. **(A)** Volcano plot of DEGs in coronavirus-associated viral sepsis from the GSE164805 dataset. Blue dots represent downregulated DEGs, red dots indicate upregulated DEGs, and gray dots denote genes without significant differential expression. **(B)** Heatmap of DEGs in coronavirus-associated viral sepsis from the GSE164805 dataset, displaying expression patterns of filtered DEGs (|log2FC| > 1, *p* < 0.05) across sample groups. Rows correspond to genes and columns represent samples, with red indicating high expression and blue low expression. Hierarchical clustering demonstrates distinct expression profiles between the viral sepsis group (left, *n* = 5) and healthy controls (right, *n* = 5), further validating intergroup transcriptional divergence.

The Limma package was utilized to conduct differential analysis on the two groups of GSE164805 dataset, with a significance threshold set at *p* < 0.05 and |logFC| > 1. As a result, a total of 15,059 differentially expressed genes were identified. Among them, there were 7,458 up-regulated genes (represented by red dots in Figure 1A) and 7,601 down-regulated genes (represented by blue dots in Figure 1A). The findings were visualized using volcano plots (Figure 1A) and heat maps (Figure 1B).

### 3.2 Identification of ferroptosis-related DEGs

**Figure 2:**
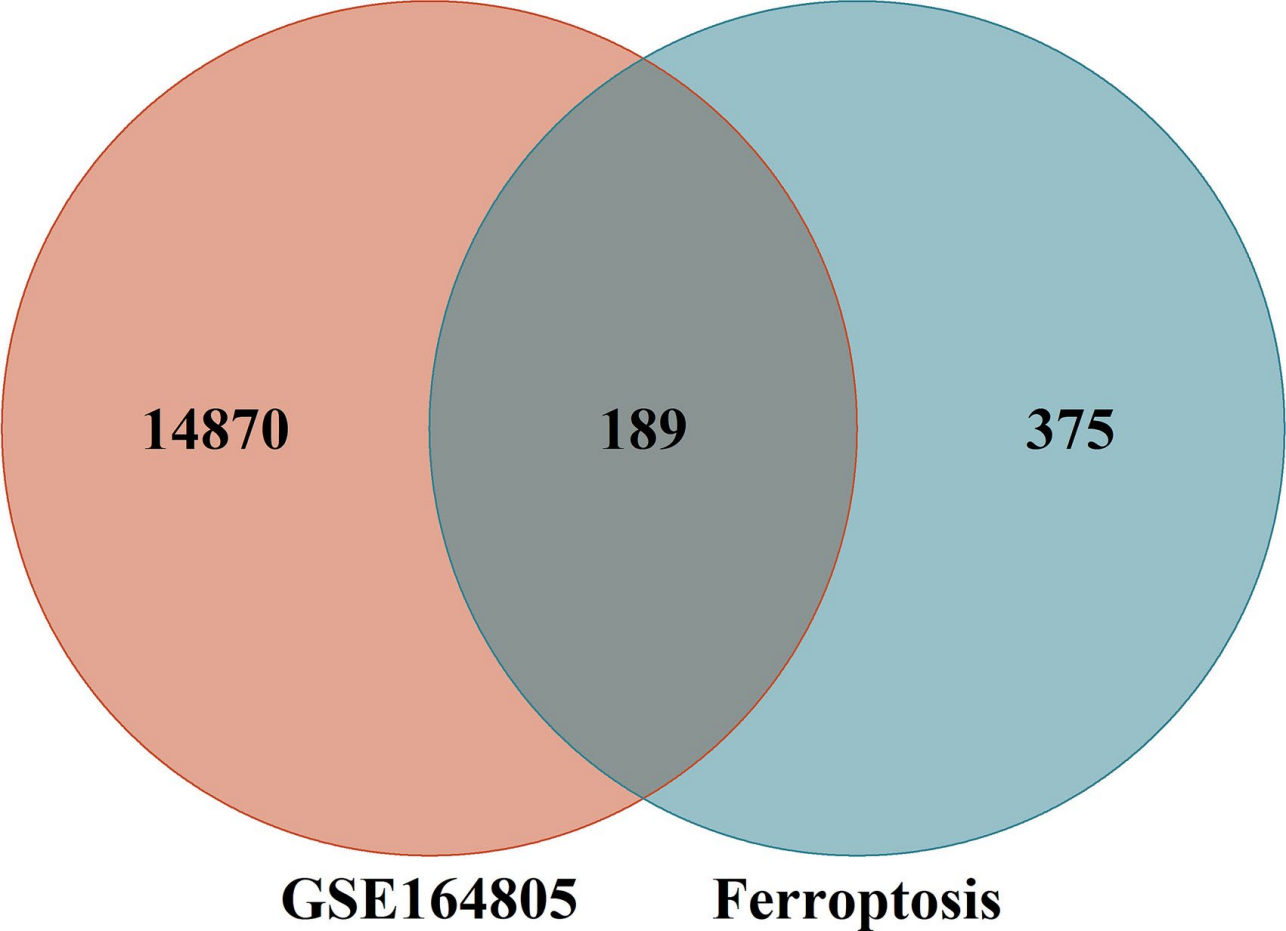
Intersection of differentially expressed genes (DEGs) in the GSE164805 and ferroptosis genes. The count on the left (14,870 genes) refers to DEGs unique to GSE164805; the count in the middle (189 genes) refers to ferroptosis-related DEGs; and the count on the right (375 genes) refers to unique to ferroptosis genes.

The FerrDb V2 database provided a total of 564 ferroptosis genes. The intersection between the aforementioned coronavirus-associated viral sepsis DEGs and ferroptosis genes resulted in 14,870 differentially expressed genes associated with coronavirus-associated viral sepsis. Among them, 189 genes were identified as differentially expressed genes related to ferroptosis in coronavirus-associated viral sepsis (Supplementary Table 1). Additionally, a total of 375 genes were classified as ferroptosis genes (Figure 2).

### 3.3 Enrichment analysis of ferroptosis-related DEGs

**Figure 3:**
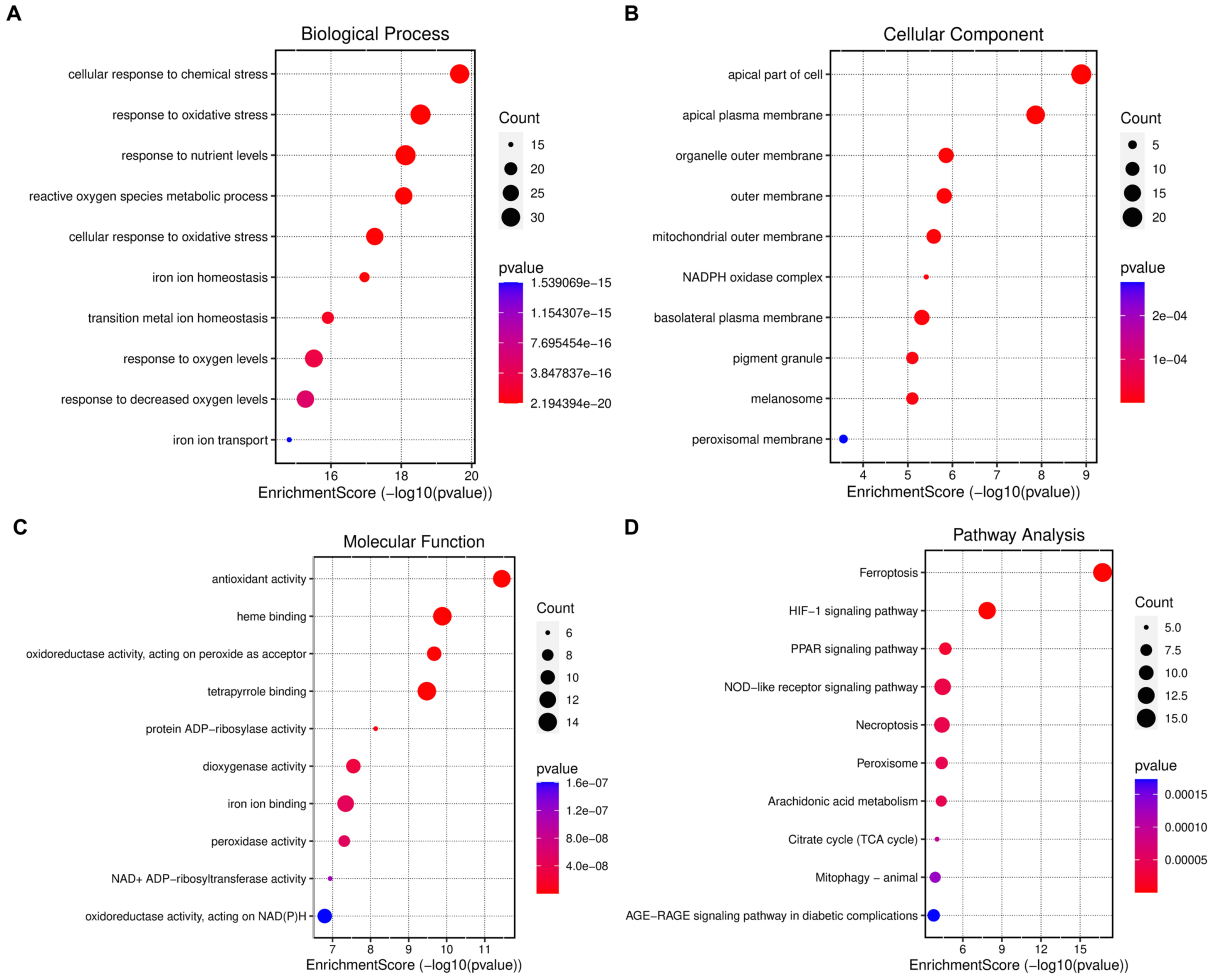
Enrichment analysis of ferroptosis-related DEGs. **(A)** Top 10 GO (gene ontology) biological processes pathway. **(B)** Top 10 GO cellular component pathway. **(C)** Top 10 GO molecular function pathway. **(D)** Top 10 KEGG pathway.

The differentially expressed genes (DEGs) associated with ferroptosis resulting from coronavirus-associated viral sepsis were subjected to analysis using Gene Ontology (GO) and Kyoto Encyclopedia of Genes and Genomes (KEGG). In the GO-BP analysis (Figure 3A), the primary pathway identified was oxidative stress. The GO-CC analysis (Figure 3B) revealed a significant enrichment of extracellular membrane-related processes. The results of the GO-MF enrichment analysis (Figure 3C) primarily indicated activation of pathways related to oxidative stress. KEGG analysis (Figure 3D) highlighted key signaling pathways including ferroptosis, HIF-1, PPAR, and NOD-like receptor signaling.

### 3.4 Landscape of immune infiltration between coronavirus-associated viral sepsis and control

**Figure 4:**
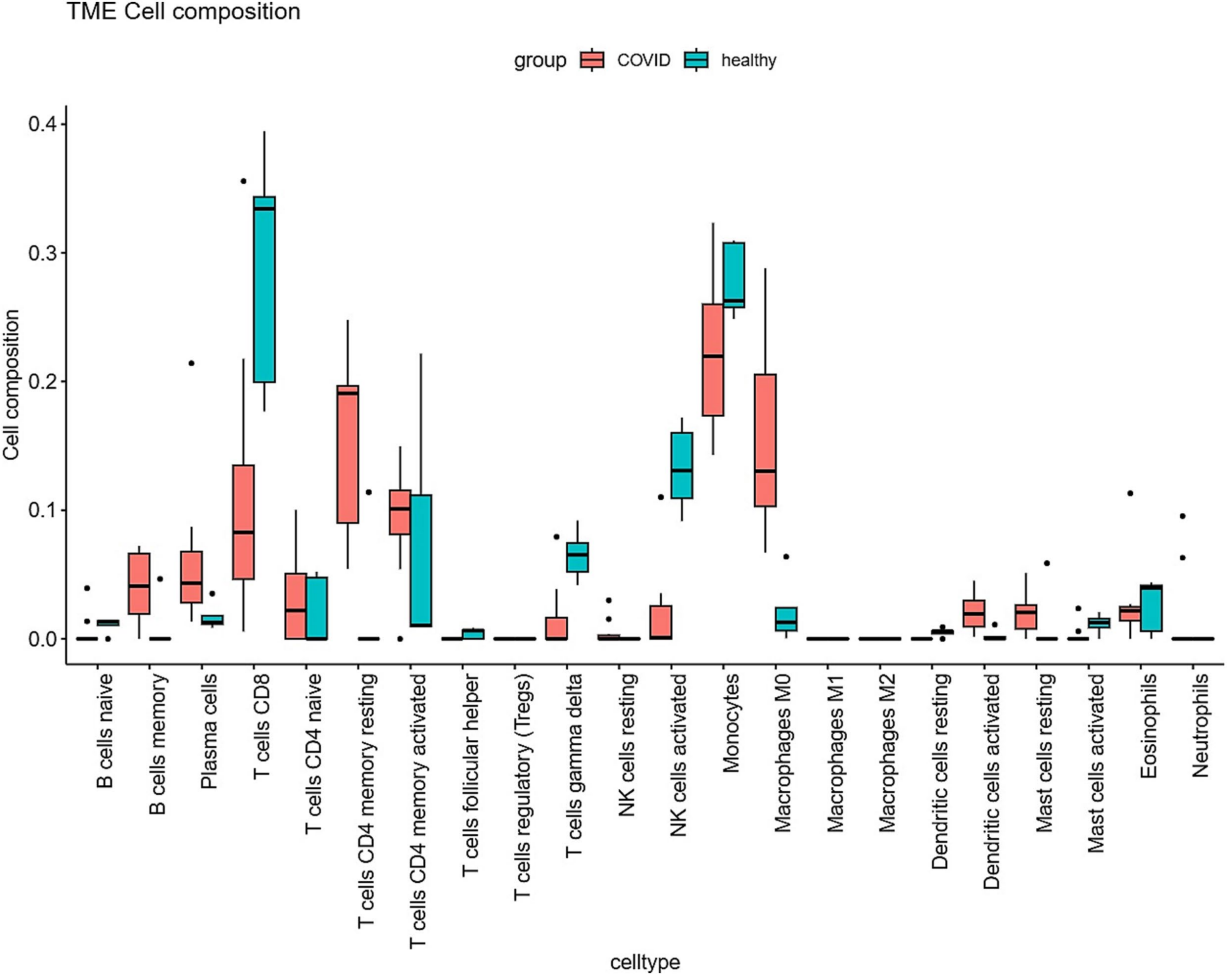
Differential expression of different types of immune cell marker expression between viral sepsis and controls.

**Figure 5:**
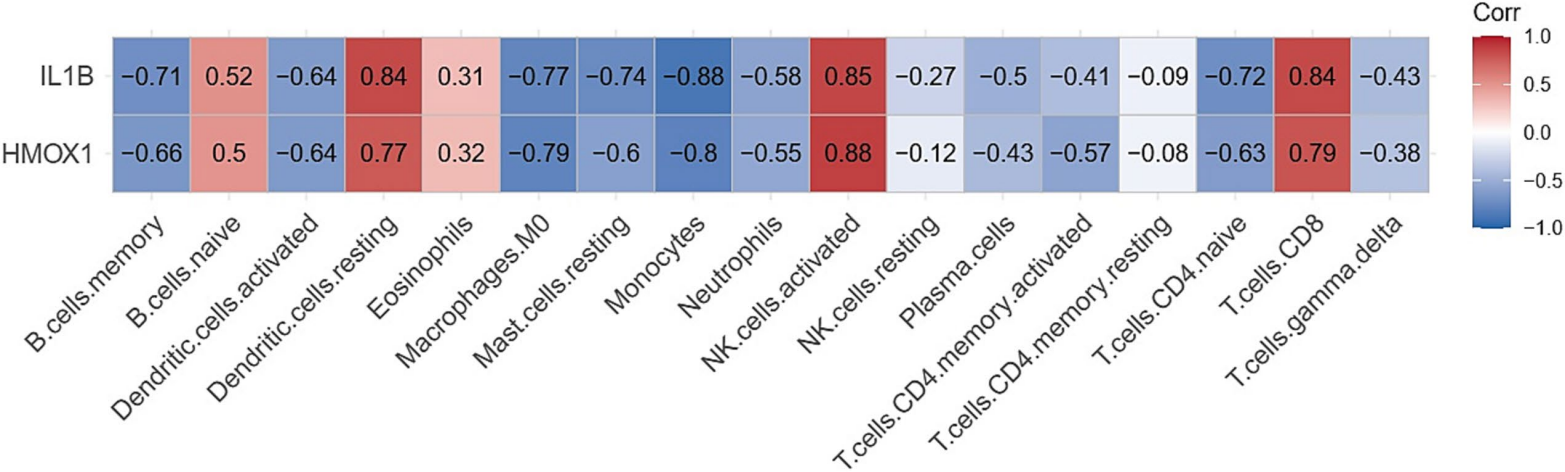
Correlation analysis between immune cell infiltration and gene expression profiles.

The Cibersort R software package was utilized to analyze immune cell infiltration in samples within the GSE164805 dataset. The box plot illustrating differences in immune cell populations revealed a higher presence of innate immune cells (such as dendritic cells, mast cells, and macrophages) in coronavirus-associated viral sepsis compared to the control group. Conversely, CD8^+^ T cells and NK cells exhibited lower levels of infiltration, as depicted in Figure 4. Furthermore, Figure 5 highlights a strong correlation between two hub genes, IL1-β and HMOX1, with the infiltration of CD8^+^ T cells and NK cells.

### 3.5 PPI network analysis

**Figure 6:**
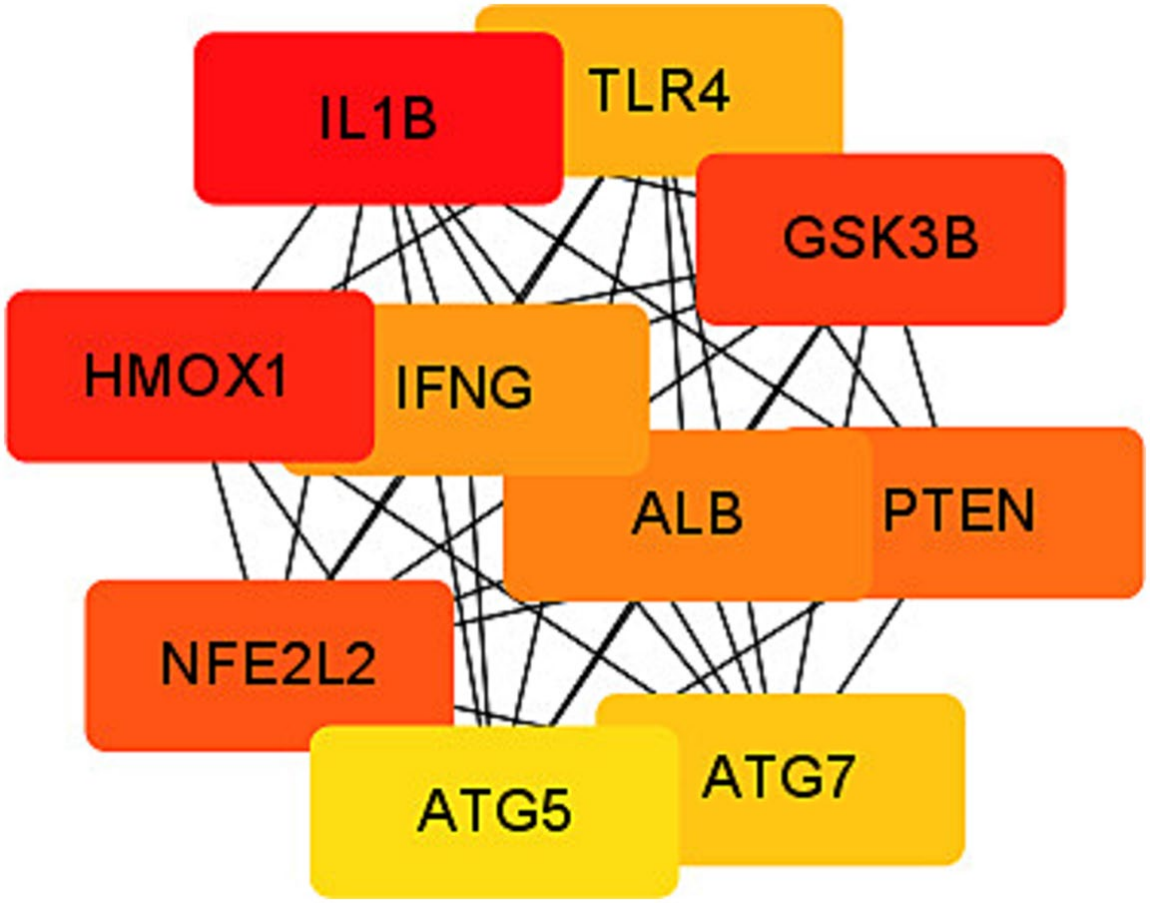
Protein–protein interaction (PPI) network of ferroptosis DEGs. Subnetwork of hub genes from the PPI network. Node colors reflect differential connectivity degrees, with red indicating higher interaction connectivity and yellow representing lower connectivity.

Among the 189 differentially expressed genes (DEGs) associated with ferroptosis in the context of coronavirus-associated viral sepsis, four of them are non-coding genes. These DEGs were imported into the STRING online database using default confidence settings (minimum interaction requirement score 0.4). Consequently, we obtained a protein–protein interaction (PPI) network comprising 185 nodes representing encoded proteins and 965 edges denoting interactions between these proteins. However, it is important to note that among these nodes, there were 11 proteins that remained unconnected to other molecules, that is, did not contribute to the formation of any molecular network (Supplementary Figure 1). To identify hub genes within this PPI network, we employed the Cytoscape CytoHubba plug-in and selected IL1-β and HMOX1 as the top two hub genes based on their highest scores according to the MCC algorithm (Figure 6).

### 3.6 Analysis of hub genes in validation sets

**Figure 7:**
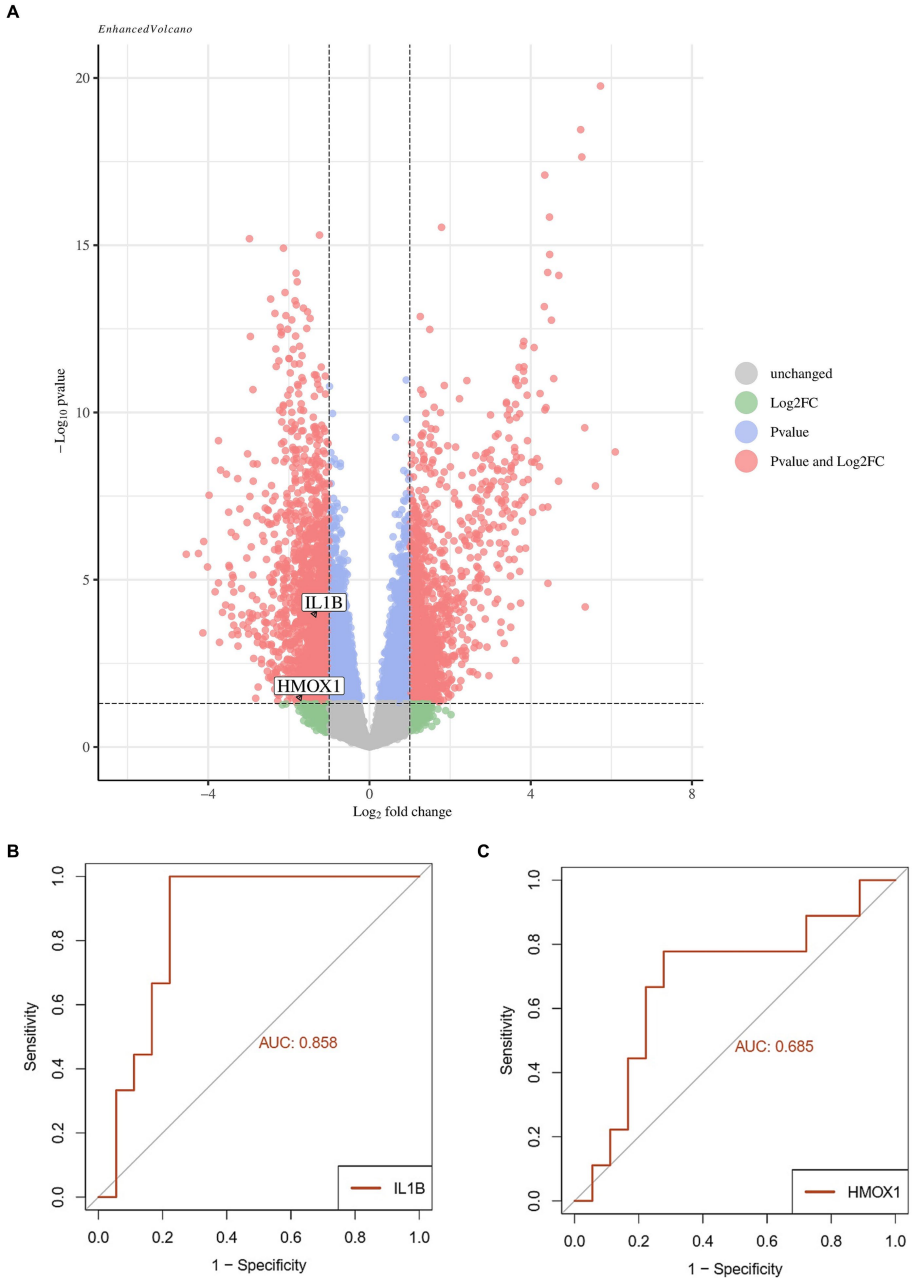
Validation analysis of key genes in the validation cohort. **(A)** Volcano plot of differentially expressed genes (DEGs) between the viral sepsis group and pathological control group in the GSE199816 dataset. Key ferroptosis-related genes (IL1B and HMOX1) are labeled, both showing decreased expression in the viral sepsis group. **(B)** Receiver operating characteristic (ROC) curve of IL1B for diagnosing viral sepsis. **(C)** ROC curve of HMOX1 for diagnosing viral sepsis.

The two screened ferroptosis hub genes IL1-β and HMOX1 were analyzed in the validation set (GSE199816). The results showed that compared to the bacterial sepsis group, the expression of ferroptosis hub genes IL1-β and HMOX1 was decreased in the coronavirus-associated viral sepsis group (Figure 7A). Further analysis of diagnostic efficacy showed that the AUROC of IL1-β and HMOX1 for the diagnosis of coronavirus-associated viral sepsis was 0.858 and 0.685, respectively (Figures 7B,C).

### 3.7 The mRNA expression levels of the hub genes of ferroptosis were determined by real-time qPCR

**Figure 8:**
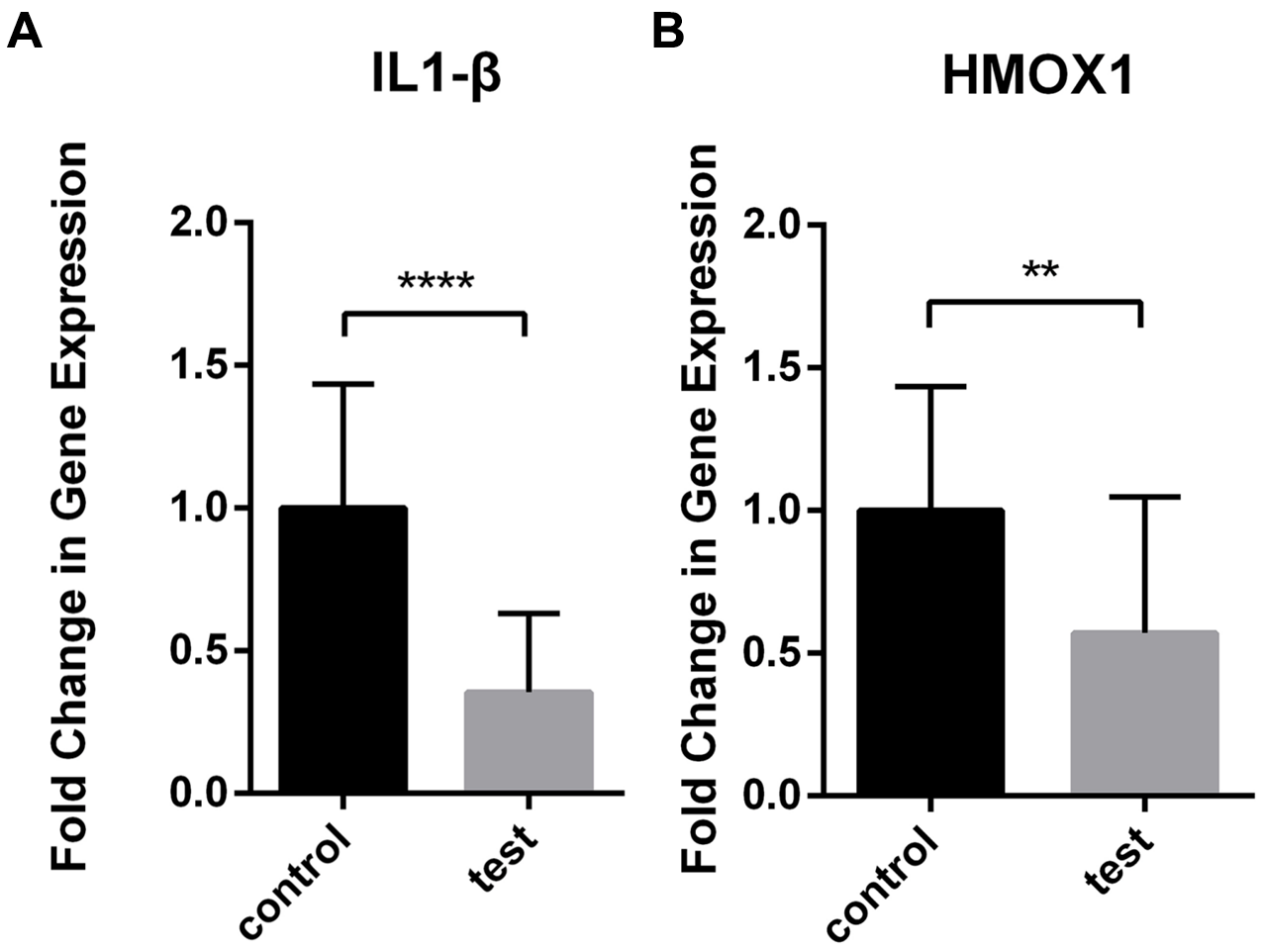
Differential expression of IL1-β and HMOX1 in viral sepsis versus healthy controls. Box plots depict mRNA levels of IL1-β **(A)** and HMOX1 **(B)** in the viral sepsis group (*n* = 20) and healthy controls (*n* = 20).

**Figure 9:**
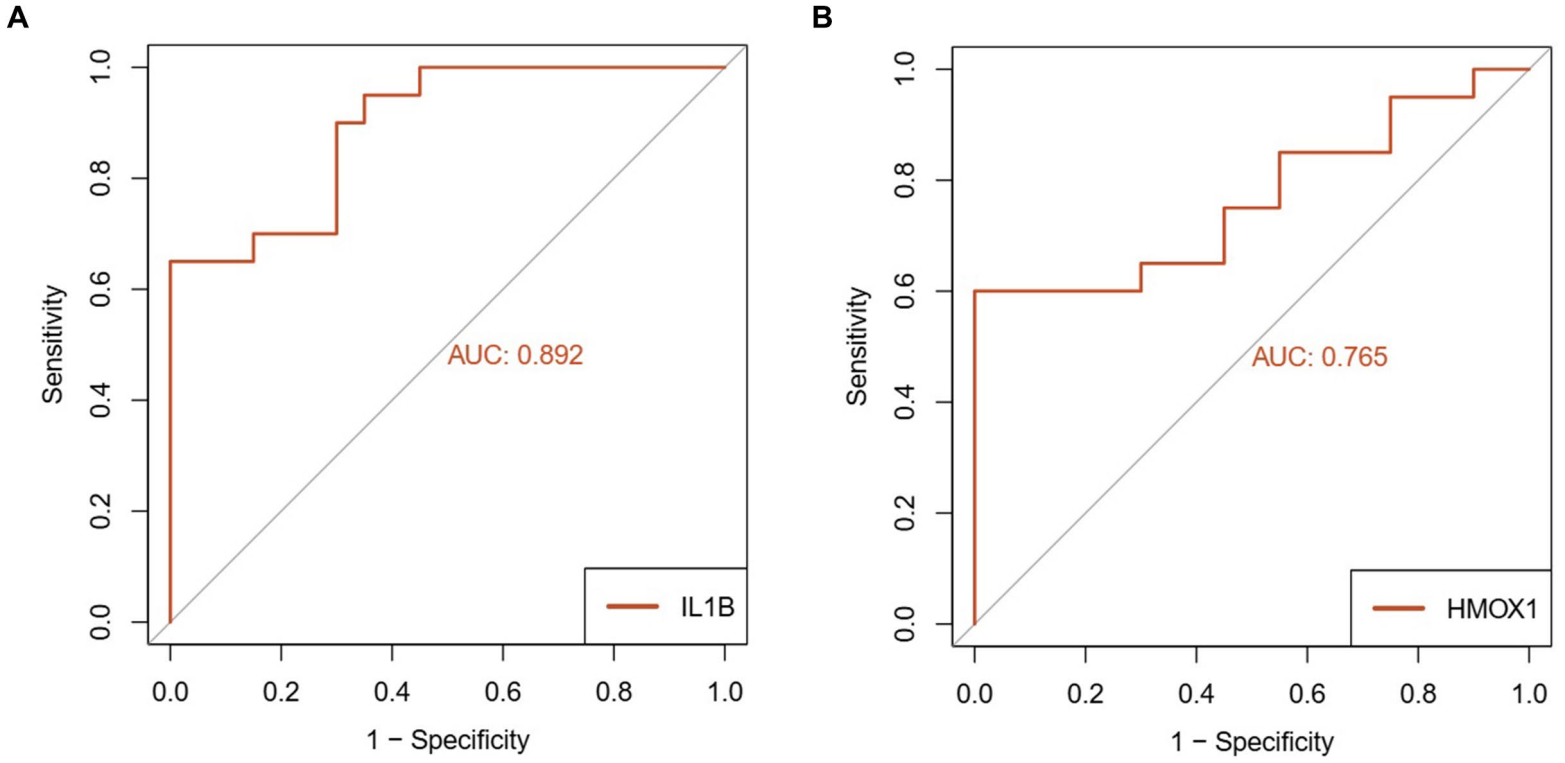
Diagnostic performance analysis of key genes in viral sepsis patients. **(A)** Receiver Operating Characteristic (ROC) curve of IL1-β for diagnosing viral sepsis. **(B)** ROC curve of HMOX1 for diagnosing viral sepsis.

The expression levels of IL1-β and HMOX1 were assessed in the test group (*n* = 20) and the control group (*n* = 20) using RT-qPCR. The results demonstrated a significant down-regulation of IL1-β (0.390 ± 0.068) and HMOX1 (0.629 ± 0.117) expressions in patients with coronavirus-associated viral sepsis compared to the control group (1.101 ± 0.107), as depicted in Figures 8A,B, respectively, with statistical significance observed for both comparisons (both *p* < 0.05). Further analysis revealed that the AUROC values for diagnosing coronavirus-associated viral sepsis based on these two hub genes were 0.892 and 0.765, respectively, as shown in Figures 9A,B.

## 4 Discussion

Viral sepsis significantly amplifies the case fatality rate of patients, particularly in those with comorbidities, the elderly population, and individuals with compromised immune systems (15). It exerts a substantial impact on patients’ outcomes and places immense strain on healthcare systems (16). Therefore, actively exploring the molecular markers of viral sepsis is imperative for early identification and development of effective treatment strategies as well as enhancement of patient prognosis (17). Iron-dependent cell death (known as ferroptosis) represents a non-apoptotic form of cell demise characterized by iron-mediated lipid peroxidation and has been implicated in various diseases including infections and inflammatory conditions (18). This study focuses on investigating differential gene expression patterns associated with ferroptosis in coronavirus-associated viral sepsis along with exploring potential signaling pathways involved to unveil prospective biomarkers and therapeutic targets.

In this study, two datasets of coronavirus-associated viral sepsis (19) were obtained from the GEO database for bioinformatics analysis, including one experimental dataset (GSE164805) and one validation dataset (GSE199816). The intersection of differentially expressed genes (DEGs) related to ferroptosis with the FerrDB V2 database revealed 189 genes that were differentially expressed and associated with ferroptosis. GO and KEGG analyses indicated that ferroptosis was primarily linked to oxidative stress, the HIF-1 signaling pathway, the PPAR signaling pathway, and the NOD-like receptor signaling pathway. These findings support previous research (20) suggesting that these pathways influence cell iron processing, regulate cell survival and death through modulation of gene expression involved in iron metabolism, thereby providing valuable insights into the molecular mechanisms underlying coronavirus-associated viral sepsis. Furthermore, studies have demonstrated that the HIF-1 signaling pathway can mediate inflammation and lead to cytokine storms associated with disease progression in coronavirus-associated viral sepsis (21). The PPAR signaling pathway has been shown to impact immune response severity and disease progression during viral sepsis (22). Our enrichment analysis suggests that targeting the PPAR signaling pathway may serve as a potential therapeutic strategy for reducing excessive inflammation and improving patient outcomes by regulating inflammatory responses in coronavirus-associated viral sepsis. Abnormalities in NOD-like receptor (NLRs) signaling pathways are associated with excessive inflammation and disease severity due to their involvement in innate immunity (23). The enrichment of NOD-like receptor signaling pathways observed in this study highlights an exaggerated immune response during coronavirus-associated viral sepsis.

We utilized the CIBERSORT algorithm to analyze the distribution of immune cells in the dataset samples. The results demonstrated that, compared to the control group, the coronavirus-associated viral sepsis group exhibited enhanced infiltration of innate immune cells, characterized by a significant increase in dendritic cell proportions (suggesting active antigen-presenting function), elevated mast cell infiltration, and macrophage enrichment (potentially linked to excessive inflammatory responses). Conversely, reduced infiltration of adaptive immune cells was observed, marked by a significant decrease in CD8⁺ T cell proportions (indicative of virus-specific T cell exhaustion, which may delay viral clearance) and diminished NK cell levels (suggesting impaired cytotoxic function). This observation is consistent with Xu et al.’s study, which reported a weakened T/B cell-specific response in severe cases, indicating an imbalance between innate and adaptive immunity (24). Further analysis demonstrated a strong correlation between the infiltration of CD8^+^ T cells and NK cells with IL1-β and HMOX1. This result aligns with our PPI network protein interaction analysis, where IL1-β and HMOX1 were identified as the top two genes with the highest screening scores. These congruent results emphasize that identifying IL1-β and HMOX1 as hub genes related to immune cell infiltration is particularly noteworthy. Notably, activated macrophages produce IL1-β as a proprotein, which has been implicated in severe COVID-19-related cytokine storms (25). Additionally, HMOX1 has been found to impact ferroptosis processes, whereby upregulation of HMOX1 can mitigate ferroptosis damage (26). Given its protective effect, targeting HMOX1 represents a promising therapeutic approach aimed at reducing inflammation and oxidative stress in coronavirus-associated viral sepsis for potential improvement of patient outcomes.

The two selected hub ferroptosis differential genes were successively analyzed in validation data sets and clinical cases, and the results showed that compared with the case control group and the healthy control group, the expression of IL1-β and HMOX1 in the coronavirus-associated viral sepsis group decreased, which was consistent with the above immune infiltration analysis results. These findings unveil distinctive molecular-level changes and immune cell responses among patients afflicted with coronavirus-associated viral sepsis. The downregulation of IL1-β and HMOX1 may be associated with the regulation of inflammatory response and oxidative stress response, while the decrease in CD8^+^ T cells and NK cells suggests immune function suppression, collectively influencing the pathophysiological process of viral sepsis. Further analysis of diagnostic efficacy showed that both of the two hub genes had high diagnostic ability for coronavirus-associated viral sepsis. Future studies could integrate these hub genes with parameters such as peripheral blood lymphocyte counts to develop effective predictive models for viral sepsis.

Despite these significant findings, there are limitations to this study. Firstly, the study heavily relies on bioinformatic analysis and only incorporates a limited number of clinical trials, thus providing evidence solely for biological mechanisms. Secondly, the sample size is relatively small and lacks large-scale clinical validation, which is crucial for confirming the diagnostic potential of the identified hub genes in ferroptosis. Finally, we fully acknowledge that viral sepsis associated with other viruses (e.g., influenza viruses, adenoviruses, etc.) may have similarities and differences in inflammatory responses, ferroptosis, and organ damage. Therefore, future studies can be further expanded to include cases of sepsis caused by different viral infections and compare studies on viral sepsis caused by cross-species viral infections, so as to reveal the similarities and differences in organ tissue and cell damage caused by different viral infections and promote more comprehensive research on viral sepsis.

In conclusion, this study has identified two hub differentially expressed genes associated with ferroptosis in coronavirus-associated viral sepsis: IL1-β and HMOX1. Functional enrichment analysis has revealed important biological processes and signaling pathways related to these two genes. Immunocell infiltration analysis has shown that innate and adaptive immune responses were dysregulated in coronavirus-associated viral sepsis, as well as decreased infiltration of CD8^+^ T cells and NK cells was closely linked to the two hub ferroptosis genes, IL1-β and HMOX1. The verification in the validation dataset and clinical case samples also demonstrated that the expressions of the hub ferroptosis genes IL1-β and HMOX1 were decreased in patients with coronavirus-associated viral sepsis, while diagnostic efficacy analysis has demonstrated their potential as diagnostic biomarkers. These findings have significantly advanced our understanding of the pathogenesis of viral sepsis, laid a foundation for further research, and provided a theoretical basis for exploring potential therapeutic targets and developing diagnostic markers.

## Data Availability

The datasets presented in this study can be found in online repositories. The names of the repository/repositories and accession number(s) can be found in the article/Supplementary material.

## Footnotes

^1^ http://www.ncbi.nlm.nih.gov/geo

^2^ http://www.zhounan.org/ferrdb/

^3^ https://string-db.org/
