# Supplementary figures and images for: Expression and diagnostic values of ferroptosis-related genes in coronavirus-associated viral sepsis
Source: Front Med (Lausanne). 2025 Apr 30;12:1496834. doi: 10.3389/fmed.2025.1496834 (PMC12074931; doi:10.3389/fmed.2025.1496834)

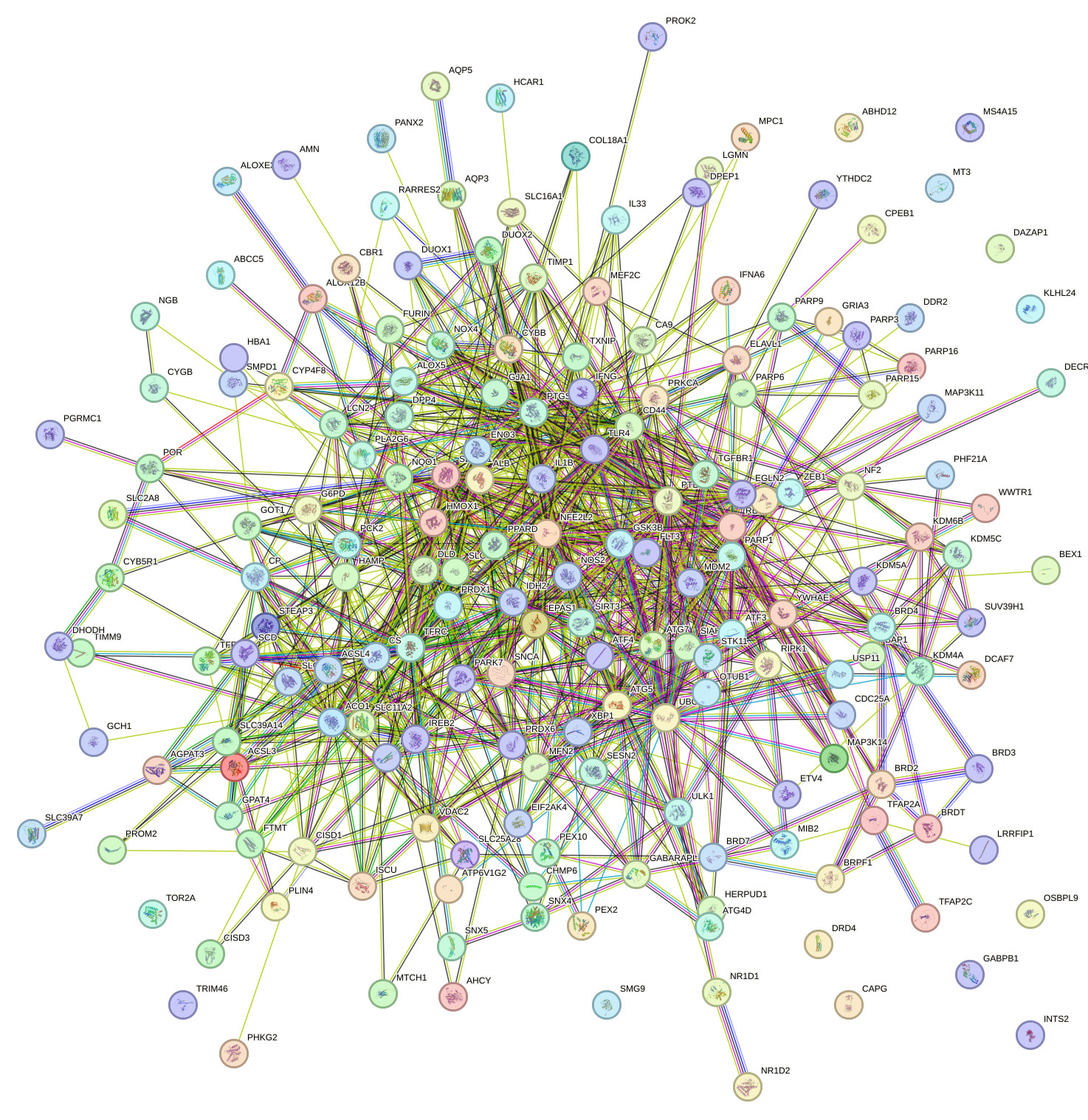

Supplement: Supplementary file 1 [file Image_1.jpeg]
